# Dog’s Discrimination of Human Selfish and Generous Attitudes: The Role of Individual Recognition, Experience, and Experimenters’ Gender

**DOI:** 10.1371/journal.pone.0116314

**Published:** 2015-02-25

**Authors:** Fabricio Carballo, Esteban Freidin, Natalia Putrino, Carolina Shimabukuro, Emma Casanave, Mariana Bentosela

**Affiliations:** 1 Canid Behavior Research Group (ICOC), Medical Research Institute (IDIM; CONICET-UBA), Buenos Aires, Argentina; 2 Instituto de Investigaciones Biológicas y Biomédicas del Sur (INBIOSUR; CONICET-UNS), Bahía Blanca, Argentina; 3 Instituto de Investigaciones Económicas y Sociales del Sur (IIESS; CONICET-UNS), Bahía Blanca, Argentina; Oregon Health and Science University, UNITED STATES

## Abstract

Discrimination of and memory for others’ generous and selfish behaviors could be adaptive abilities in social animals. Dogs have seemingly expressed such skills in both direct and indirect interactions with humans. However, recent studies suggest that their capacity may rely on cues other than people’s individual characteristics, such as the place where the person stands. Thus, the conditions under which dogs recognize individual humans when solving cooperative tasks still remains unclear. With the aim of contributing to this problem, we made dogs interact with two human experimenters, one generous (pointed towards the food, gave ostensive cues, and allowed the dog to eat it) and the other selfish (pointed towards the food, but ate it before the dog could have it). Then subjects could choose between them (studies 1-3). In study 1, dogs took several training trials to learn the discrimination between the generous and the selfish experimenters when both were of the same gender. In study 2, the discrimination was learned faster when the experimenters were of different gender as evidenced both by dogs’ latencies to approach the bowl in training trials as well as by their choices in preference tests. Nevertheless, dogs did not get confused by gender when the experimenters were changed in between the training and the choice phase in study 3. We conclude that dogs spontaneously used human gender as a cue to discriminate between more and less cooperative experimenters. They also relied on some other personal feature which let them avoid being confused by gender when demonstrators were changed. We discuss these results in terms of dogs’ ability to recognize individuals and the potential advantage of this skill for their lives in human environments.

## Introduction

Some animals have the capacity to identify and remember stable behavioral dispositions in other individuals [1–3]. This ability, which has been called “reputation attribution”, is probably adaptive in helping to predict others’ behavior, and, authors agree, is particularly relevant in the context of cooperative exchanges [4–7]. In the case of humans, the ability to attribute and remember others’ reputation, it is argued, has played a major evolutionary role in stabilizing reciprocally beneficial relations [8, 9]. Moreover, its comparative study promises to deliver insights in terms of convergent and divergent selection forces driving social cognition in different species [10–12].

The attribution of reputation can be based on direct exchanges with target individuals or indirectly by observing third-party interactions [13]. Some authors argue that reputation tracking requires cognitively demanding abilities, such as individual recognition and a detailed memory of behaviors and exchange results [8, 14]. Limitations on these cognitive abilities and their consequences on cooperative tasks have been identified in different species including humans [8, 15–18]. We here explore the ability to attribute reputation to people in domestic dogs (*Canis familiaris*).

The increasing interest in dogs’ social and cognitive abilities has been mainly driven by the recognition that their long domestication process (as long as 15000–33000 years) [19, 20] has caused morphological, physiological and behavioral changes that may have allowed them to adapt to human environments [21, 22]. Moreover, it has been claimed that some of dogs’ specific features and social abilities show signs of convergent evolution with humans [10, 23]. In addition, domestic dogs live in intimate contact with people throughout their lives, thus having plenty of instances to learn to predict people’s behavior and the consequences of interacting with them [22, 24]. Indeed, most relevant resources for domestic dogs, such as food, water and shelter, come from people. This dependence would explain their acute sensitivity to human communicative cues such as pointing gestures, prohibitive signals, and even emotional expressions [25–30].

In this investigative context, researchers have increasingly invested effort in studying dogs’ abilities to discriminate and remember people’s cooperative dispositions [31, 32]. There is evidence that dogs can develop a preference for a person after watching her having a cooperative interaction with a third individual. In the basic setup, the dog watches a scene in which there are two human demonstrators who have food, and a third individual (the beggar) that interacts with them [33, 34]. The beggar asks each of the demonstrators for food in sequential turns. The “generous” demonstrator always gives food to the beggar, whereas the “selfish” demonstrator always withholds it. After the demonstration phase, the dog is allowed to choose between the demonstrators. It has been found that dogs preferentially approach the generous over the selfish person [33, 34]. Not only that, but even after making demonstrators’ actions the same, dogs can discriminate between them just based on the beggar’s (dis)satisfaction reactions during demonstrations [31]. Nevertheless, Freidin and collaborators found that if demonstrators swapped places after demonstrations but before the dog was allowed to choose (with the aim of controlling for local enhancement), dogs’ preference was not different from chance [31]. That is to say that their choices between demonstrators were not based on remembering personal cues, but, instead, on recalling the place where the beggar had shown positive reactions. Results from Nitzschner et al. [35] are consistent with this interpretation. These authors replicated the procedure done by Marchsal-Pescini et al. [34], but added a control group in which, out of the dog’s sight, the demonstrators swapped places before the animal could choose [35]. In this control group, dogs did not show any preference between demonstrators. In contrast, Kundey et al. found that dogs preferred to approach the generous demonstrator even after demonstrators exchanged places before the choice test [33]. It is important to note that the number of demonstration trials was higher in the study by Kundey et al. [33] than in the studies by Freidin et al. [31] and Nitzschner et al. [35]. This suggests that dogs rapidly detect generous and selfish attitudes in people, but that they may require more experience to individually recognize each demonstrator and associate her with the corresponding attitude.

In sum, these studies taken together indicate that dogs can attribute reputation indirectly after observing interactions among strangers. However, they seemingly rely on multiple cues (not necessarily from the person) and need several observations to perform above chance levels.

Unlike the indirect attribution of reputation, judgments based on direct encounters would be less demanding in cognitive terms [36]. In studies in which direct reputation tracking has been assessed in dogs, typically, two human strangers interact with the dog in turns and later the dog is allowed to choose between them. Petter et al. designed a protocol in which two experimenters, one cooperative and the other deceitful, stood behind one of two containers, pointed at it and verbally encouraged the dog to approach it [32]. The cooperative experimenter always stood behind the container with food, whereas the deceitful experimenter always stood behind the empty container. In a different study, they did the same protocol but used boxes of two different colors instead of different experimenters. In both procedures (with the human experimenters and with the boxes), dogs learned to approach the container with food over the empty container. Interestingly, they did not develop a preference for the cooperative over the deceitful experimenter as evidenced in the choice test, although they did show a preference for the “cooperative” over the “deceitful” box [32]. According to the authors, the most parsimonious explanation for these findings would presume the formation of a simple association of the human and the box cues with the different outcomes (i.e., food vs. no food). Possibly, the difference in the choice test between people and boxes would be a consequence of the discrimination between people being more difficult than the discrimination between boxes of different color. This last idea could be tested by facilitating the discrimination between people, for example, by using experimenters of different gender given the fact that dogs may react differently to men and women [37].

In a similar line of research, McMahon et al. showed that dogs preferred to approach a human informant who pointed towards the box under which there was food over a non-informant demonstrator who turned her back to the dog when the animal had to guess where the bait was [38]. This finding may suggest that dogs recognized who had a better disposition to help. However, these conclusions need to be taken with caution because McMahon et al.’s results could be explained by the fact that, when searching for food, untrained dogs tend to approach people facing them relative to those who give their back [39]. A similar issue emerges in the study done by Petterson et al. [40]. These authors used a pointing task in which the generous experimenter made a pointing gesture towards the bowl with food, whereas the selfish experimenter made a prohibitive gesture and verbalization. Unfortunately, these authors used a cue, the word “no” in a prohibitive tone, which is usually learned by dogs during their ontogeny in human contexts. In this sense, its use by the selfish but not the generous experimenter could be the main cue driving subjects’ inferior performance in the prohibitive relative to the cooperative context.

In synthesis, the studies reviewed above provide suggestive but not conclusive evidence that domestic dogs are capable of attributing reputation to people whom they directly interact with. The main issue with previous findings is that there are alternative explanations that have not been controlled for. These untested possibilities leave many questions open relative to the limits of dogs’ abilities to discriminate and track people’s cooperative dispositions, and the circumstances which facilitate their use.

The goal of the research presented here was to further study dogs’ ability to attribute reputation to people after direct interactions with them. We first used an object choice task in which experimenters would make a proximal pointing gesture towards a bowl with food (as opposed to an empty bowl). There were training trials with a generous experimenter and other with a selfish experimenter. Whereas the “generous” experimenter allowed the dog to eat from the bowl after the pointing gesture, the “selfish” experimenter ate the food just before the dog’s approach thus precluding its access to it. Finally, we did choice tests in which the two experimenters were simultaneously present, and the dog was allowed to approach them so that we could measure its preference. Our main interest was in exploring which human cues the dogs used to discriminate between experimenters. Given the fact that dogs have been shown to react differently to men and women [37], we tested for the effect of the experimenters’ gender on dogs’ discrimination performance. In study 1, we did the task with same-gender experimenters, and, in study 2, we did a similar protocol using experimenters of different gender. Last, we tested whether dogs would make systematic errors based on the experimenters’ gender by changing the experimenters (and introducing new ones) between the training and the choice phase in study 3.

## Study 1

The goal of study 1 was to evaluate dogs’ behavior towards “generous” and “selfish” experimenters (hereafter Es or E for the singular form), defined as who gave or withheld food from the dog in a communicative context (a pointing task). It is worth noting that although we operationally defined “selfish” and “generous” in relation to Es’ food sharing behavior, both Es acted as complex stimuli whose behavior also varied in other features such as their use of ostensive cues (see more details in the Procedure subsection below).

In study 1, Es were either two women or two men for any given subject.

### Method

#### Ethical statement

In Argentina there is no special approval required for the use of dogs in studies of social behavior and cognition in which there are no invasive or stressful manipulations. In any case, we consulted the Institutional Committee for Care and Use of Experimental Animals (CICUAL) of the Veterinary Sciences School, University of Buenos Aires. This study was carried out in strict accordance with the ethical standards of the CICUAL and complied with the current Argentine law of animal protection (Law 14346). All owners expressed their consent for the participation of their dogs in this study.

#### Subjects

We evaluated 19 naïve dogs between 1 and 10 years old. Seven dogs were discarded: three because they showed signs of fear, three due to interruptions by the owner, and one due to technical problems with the video-camera. The definitive sample had 12 subjects of different breeds (1 Boxer, 1 Weimaraner, 1 Border Collie, 1 Golden Retriever, 1 Beagle, 2 German Shepherds and 5 mixed breeds), 7 females and 5 males. Mean (±1 SD) age was 3.60 (2.44) years old. All animals were domestic pets living with their owners and none of them had any training experience. To make sure that the dogs would be motivated to do the task for food, we asked owners not to feed their pets for at least 3 h before our arrival. Subjects had free access to water throughout the sessions (see Table 1 for details, such as age and sex, of the individual subjects).

**Table 1:** Subjects’ details and choices in the test phase.

Study	Breed	Sex	Age	Size	Owners’ Gender	Generous Experimenters’ Gender	Test 1	Test 2
1	Mix Breed	M	3	Medium/Small	F & M	F	G	G
1	Mix Breed	M	4	Big	F & M	F	S	G
1	Boxer	F	1,5	Medium/Small	F & M	F	G	-
1	Mix Breed	F	7	Big	F & M	F	S	G
1	Mix Breed	F	8	Big	F & M	F	S	S
1	Weimaraner	F	2	Big	M	M	S	S
1	Mix Breed	F	4	Medium/Small	F & M	M	-	G
1	Golden Retriever	F	1	Big	F & M	M	G	G
1	Border Collie	F	2	Big	M	F	S	G
1	German Sheperd	M	4	Big	F & M	M	G	G
1	Beagle	F	9	Medium/Small	F & M	M	G	G
1	German Sheperd	M	1	Big	M	M	G	G
2	Mix Breed	M	2	Medium/Small	F	M	G	G
2	Shih Tzu	M	2	Medium/Small	F & M	M	S	G
2	Mix Breed	M	6	Big	F & M	M	G	S
2	Mix Breed	F	1,5	Medium/Small	M	M	S	G
2	Golden Retriever	F	7	Big	F & M	M	S	G
2	Golden Retriever	F	7	Big	M	M	G	G
2	German Sheperd	F	5	Big	F & M	M	G	-
2	Boxer	F	4	Medium/Small	F	M	G	G
2	Mix Breed	M	4,5	Big	F & M	F	G	S
2	Border Collie	F	8	Big	F & M	F	G	S
2	Golden Retriever	M	6	Medium/Small	F & M	F	G	S
2	Mix Breed	F	5,2	Medium/Small	F & M	F	G	S
2	German Sheperd	F	4	Big	M	F	G	G
2	Golden Retriever	M	6	Big	F & M	F	S	G
2	Mix Breed	F	7	Medium/Small	F & M	F	G	S
2	Mix Breed	F	9	Medium/Small	F & M	F	G	S
3 Experimental	Mix Breed	M	5	Medium/Small	F	F	G	-
3 Experimental	Mix Breed	M	5	Medium/Small	F & M	F	S	-
3 Experimental	Golden Retriever	M	2	Medium/Small	F & M	F	G	-
3 Experimental	French Bulldog	M	4	Medium/Small	F & M	F	S	-
3 Experimental	Bichón Frissé	M	5,5	Medium/Small	F & M	F	S	-
3 Experimental	Boxer	M	1	Big	F & M	F	G	-
3 Experimental	Pitbull	F	1	Big	F & M	F	S	-
3 Experimental	Pitbull	M	2,5	Big	F & M	F	S	-
3 Experimental	German Sheperd	M	7	Big	F & M	F	S	-
3 Experimental	Caniche Toy	M	3,5	Medium/Small	F & M	F	G	-
3 Experimental	Siberian Husky	M	8	Big	F & M	F	G	-
3 Experimental	Rottweiler	F	5	Big	F & M	F	S	-
3 Control	Mix Breed	F	3	Big	F & M	F	S	-
3 Control	Mix Breed	M	5	Medium/Small	F & M	F	G	-
3 Control	Belgian Sheperd	M	5	Medium/Small	F & M	F	G	-
3 Control	Mix Breed	F	4	Big	F & M	F	G	-
3 Control	Mix Breed	F	7	Medium/Small	F & M	F	G	-
3 Control	Caniche Toy	M	3	Medium/Small	F	F	G	-
3 Control	Setter	F	8	Medium/Small	F & M	F	G	-
3 Control	Border Collie	M	3,2	Medium/Small	F & M	F	G	-
3 Control	Boxer	M	3,2	Big	F & M	F	G	-
3 Control	Golden Retriever	F	5	Medium/Small	F & M	F	S	-
3 Control	Golden Retriever	M	8	Big	F & M	F	G	-

Note: Generous Experimenter (G); Selfish Experimenter (S). Female (F); Male (M).

#### Apparatus

**Fig 1:**
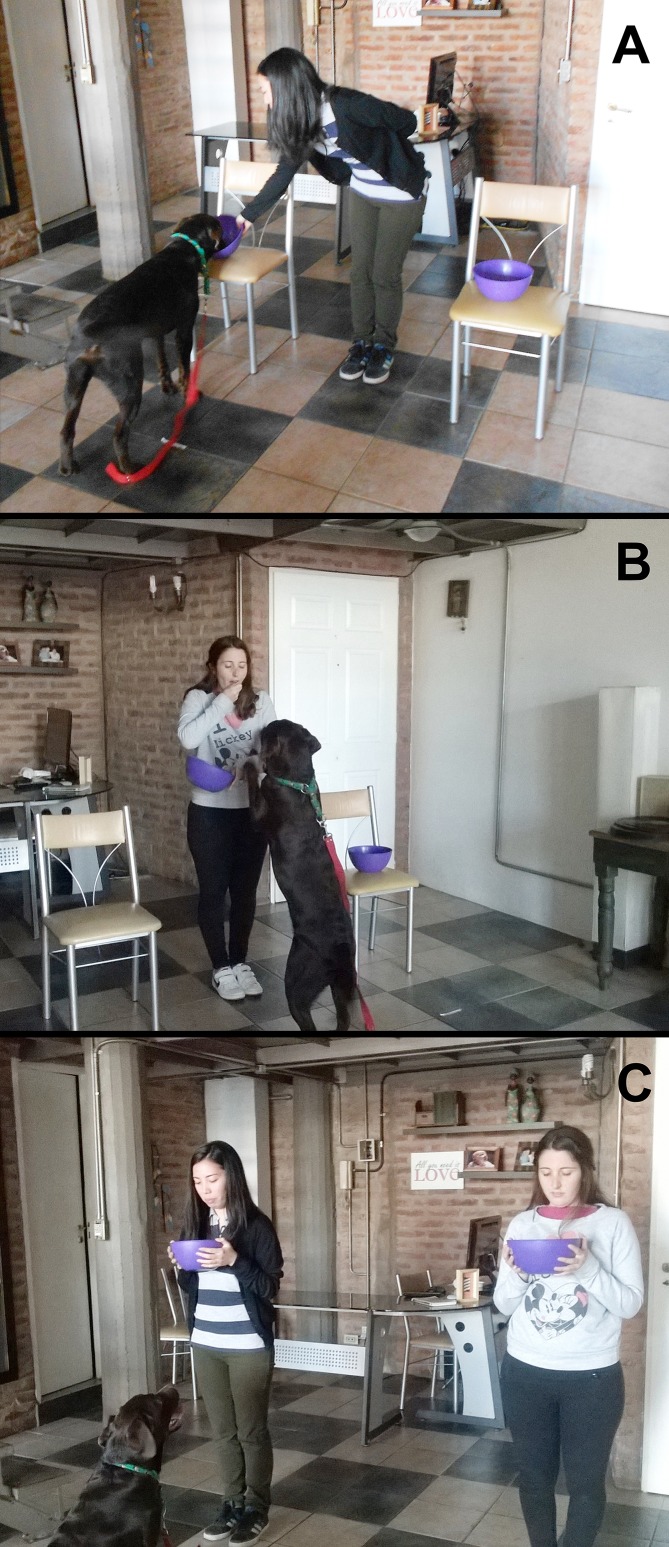
Photos of the experimental set up and procedure. (A) The generous experimenter is letting the dog eat from the baited bowl. (B) The selfish experimenter is eating the food in front of the dog. (C) Experimental set up in choice tests; the dog is approaching the experimenter on the right. All persons that appear in this figure have given written informed consent, as outlined in the PLOS consent form, to publication of their photograph.

Dogs were evaluated in their homes or in a canine day care facility familiar to them. The experimental setup consisted of two chairs placed 75 cm apart from each other, and two identical opaque bowls (20 cm diameter, and 8 cm height) in which we presented food to the animals. We used tape to draw a 50-cm perimeter on the floor around each chair to delimitate the choice area for choice tests. The dog was held by the handler 1.5 m from the E. A camera SONY DCR-SR88 was placed on a tripod behind and above the handler to video-tape the trials (see Fig. 1). Throughout the manuscript, when we refer to “food” or “reward”, we meant pieces of baked chicken which were the only food reinforcement used in these protocols. To control for olfactory cues, we hid pieces of food under a double bottom in each bowl and both containers were greased with abundant chicken. All persons that appear in Fig. 1 have given written informed consent, as outlined in the PLOS consent form, to publication of their photograph.

#### Procedure

**Fig 2:**
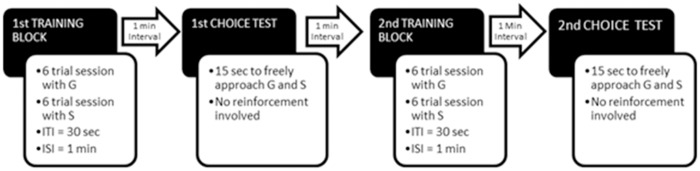
Experimental procedure in studies 1 and 2 (study 3 only involved the first training block and the first choice test). Note: G: generous experimenter; S: selfish experimenter; ITI: inter-trial interval; ISI: inter-session interval.

Fig. 2 shows the sequence of the experimental protocol. Each dog began with pre-training trials and then received two blocks of training sessions. After each block, there was a choice test. Each block of training sessions comprised a session with the generous experimenter (G) and a session with the selfish experimenter (S). Each session consisted of 6 trials; therefore, each dog received 24 training trails in total. In training trials there was only one E present (either G or S). For choice tests, both Es were simultaneously present and the dog could freely approach them. The inter-trial interval (ITI) was set at 30 sec and intervals between sessions (ISI), between blocks and between sessions and choice tests were set at 1 min.

**Pre-training**: The purpose of pre-training was to show dogs that there could be food inside the bowls so that they had an incentive to approach them. After a period of familiarization with the Es’ presence (3–5 min), G placed a bowl with food over the seat of each chair and left the scene. The handler then led the animal towards each bowl, and let the dog eat. We did two pre-training trials, and 30 sec after the end of the second one, the first training block began. Dogs that showed no interest in eating from the bowls were discarded from the sample because of their lack of food motivation.

**First training block**: The first block of sessions started with training trials by G for half of the subjects and with training trials by S for the other half. For any given subject, G and S were experimenters of the same gender unknown to the dogs before the study (for half of the dogs, Es were two women, and for the other half, Es were two men). The handler’s gender was counterbalanced across dogs as well.

Any block comprised six trials with G (called a session), and six trials with S (another session). In the beginning of any training trial, out of the dog’s sight, the corresponding E hid a piece of food in one of the two bowls. Then, the E walked with the two bowls (one with food and the other empty) towards the chairs, took her/his position in between them, and left simultaneously one bowl on the right seat and the other bowl on the left seat. The side in which the bowl with food was placed (left or right) was randomized in each trial with the restriction that we did not use the same side in more than two consecutive trials. Afterwards, the E did the pointing gesture towards the bowl with food, and the dog was released to find the reward. We used a static proximal pointing gesture as signaling cue. The E pointed with the arm and the index finger extended towards the correct bowl, with the tip of the finger less than 50 cm away from the cup, and stayed in that position until the dog made a choice between the two bowls (see Fig. 1A). The first bowl that the dog approached with its muzzle at less than 10 cm was registered as the chosen bowl.

In training trials with G, before the pointing gesture, the E looked at the dog’s face and called it by its name in a positive manner until catching its attention. Then, G did the pointing gesture, while also giving ostensive cues: she/he alternated the gaze between the dog and the food bowl and said in Spanish “Mmm… look inside, it’s so yummy, look inside, it’s so yummy!” using a positive intonation. The handler then released the dog. If the dog chose the bowl with food, it was allowed to eat it. If the dog chose the empty bowl, G pointed again towards the correct bowl until the dog approached it and ate the food (i.e., the dog always ate from the bowl in training trials with G). While it was eating, the dog was verbally reinforced by G who said in Spanish “Very good, very good!”, though there was never physical contact directed from the Es to the dogs. After the dog ate, the handler took the subject to the starting position again, and the following trial with G began after 30 sec. Once all six trials with G were done, there was a 1-min interval until either the session with S or the choice test began.

The beginning of trials with S was identical to that of trials with G. Once the E was in between the chairs and with the bowls in place, the protocols in training trials with G and S diverged. Unlike trials with G, S looked at the dog, called it by its name in a neutral tone of voice until catching its attention (a maximum of 3 calls) and did the pointing gesture towards the bowl with food without saying anything or alternating the gaze between the bowl and the dog. The handler then released the dog and, if it approached the correct bowl, S quickly took the food from the bowl, showed it to the animal, ate it in front of it, and let the subject see the empty bowl (see Fig. 1B). If the dog chose the empty bowl, S did the pointing gesture towards the correct bowl again until the dog chose it and then S ate the food in front of the dog as described. After that, the handler led the dog to the starting position, gave the subject a piece of food, and the following trial with S began after 30 sec. Feeding by the handler in training trials with S was implemented to equate the amount of reinforcement in sessions with G and S. Once the six trials with S were done, there was a 1-min interval until either the session with G or the choice test followed.

In the case that a dog did not approach any bowl within 15 sec since the beginning of the pointing gesture in any training trial (be it with G or with S), we registered a “no-choice” response. After a no-choice response, the handler gently led the subject towards the E, thus encouraging the dog to choose in order to be exposed to the corresponding E’s attitude.

**First choice test**: One min after the end of the first training block (i.e., after a session with G and a session with S), we did a preference test. For this test, chairs were removed from the scene, and G and S stood approximately where the chairs had been (though a bit further apart, at a distance of 1.5 m). The handler held the dog at the starting position, 1.5 m from the middle point in between G and S (see Fig. 1C), for about 2 s to make sure that the animal had time to observe the scene before it was released to choose. We considered a “choice response” when the dog approached one of the Es with its head facing him/her at distance of less than 50 cm according to the tape marks on the floor. If the dog did not make a choice within 15 sec since released, we registered a “no-choice” response. Throughout the choice test, G and S stood steadily and quietly, constantly looked at the dog, and gave no food reinforcement at all.

The side in which G and S stood in the choice test (left or right) was counterbalanced between the first and the second choice test for each dog, and it was also counterbalanced between subjects (i.e., for half of the dogs, the position of G was left in the first choice test, whereas for the other half, it was right).

**Second training block and second choice test**: One min after the end of the first choice test, the second training block began. This block of sessions was exactly the same as the first training block. Besides, it was also followed by a choice test which was exactly the same as the first choice test, with the exception that G and S stood on opposite sides relative to those occupied in the first test.

#### Data analyses

We used SONY Vegas 11 Pro video editor to process all recordings. In the three studies reported here, we measured the same four dependent variables: In training trials, we measured the latency to choose a bowl (hereafter referred as “latency”) and the number of correct choices (hereafter referred as “performance”). In choice tests, we measured the first choice (hereafter referred as “choice”) and the time in proximity to each E.

“Latency” was defined as the time (sec) elapsed since the start of the pointing gesture until the moment the dog had its muzzle at 10 cm from any bowl. In 30% of training trials, latencies were also measured by a second rater. The correlation between raters’ measures was very high (Pearson correlation coefficient = 0.96). Latency data were not normally distributed, so we used non-parametric statistics to analyze them. We compared cumulative (across trials of the same session) latencies in trials with G against trials with S using *Wilcoxon Matched Pairs* tests. To evaluate the influence of the Es’ gender on latencies, we used *Mann-Whitney U* tests with gender as the grouping factor. To analyze “performance” in training trials, we counted the number of correct responses in each session, that is, the number of times that the dog’s first approach was directed towards the bowl with food. This variable was normally distributed so we used *t* tests to compare average correct responses against the 0.50 chance level, and to compare dogs’ performance in sessions with G against sessions with S.

In choice tests, we registered two variables: 1) the first choice; and 2) the overall time in proximity to each E. In other words, dogs’ preferences were assessed by the animal’s first approach to either G or S, and by the time they spent in proximity to each E during the whole test. To establish a dog’s first choice, we considered which E the dog approached first with its head facing him/her at distance of less than 50 cm according to the tape marks on the floor. All choices were independently codified by two of the authors (FC and MB); in two cases of disagreement, we used the evaluation of an assistant who was unfamiliar with the experimental design. To determine if dogs chose G (over S) above the 0.50 chance level, we used binomial tests. Finally, to measure “time in proximity”, we counted the number of sec a dog spent less than 50 cm apart from each E during the entire choice test. To measure this variable, we registered the dog’s position in each of the 45 video frames comprised in the 15 sec that the choice test lasted (i.e., 3 frames per sec; we presented this measure in sec in the text). We used parametric statistical analyses (ANOVAs and *t* tests) for this variable since it was normally distributed.

In studies 2 and 3, we did the same statistical analyses reported for study 1, with the following exceptions: 1) in study 2, we used chi-square tests to assess dogs’ choices as a function of whether the E was female or male; 2) in study 3, we had two independent groups so we used *Mann-Whitney U tests* to compare the latencies between these groups; and 3) in study 3, we used a repeated measures ANOVA to compare subjects’ performance in training trials of the two independent treatments.

We also checked for possible effects of subjects’ age (by dividing the sample in each study by the median age and comparing the youngest against the oldest half) and body size (small-medium vs. large) on all four dependent variables across studies. Age and size were never significant for any variable (*Mann-Whitney U tests*, all *p*s > 0.05).

The α-value was set at 0.05 and all tests were two tailed. We used SPSS statistical package v. 19 for the analyses.

### Results

#### Latencies in training trials

**Fig 3:**
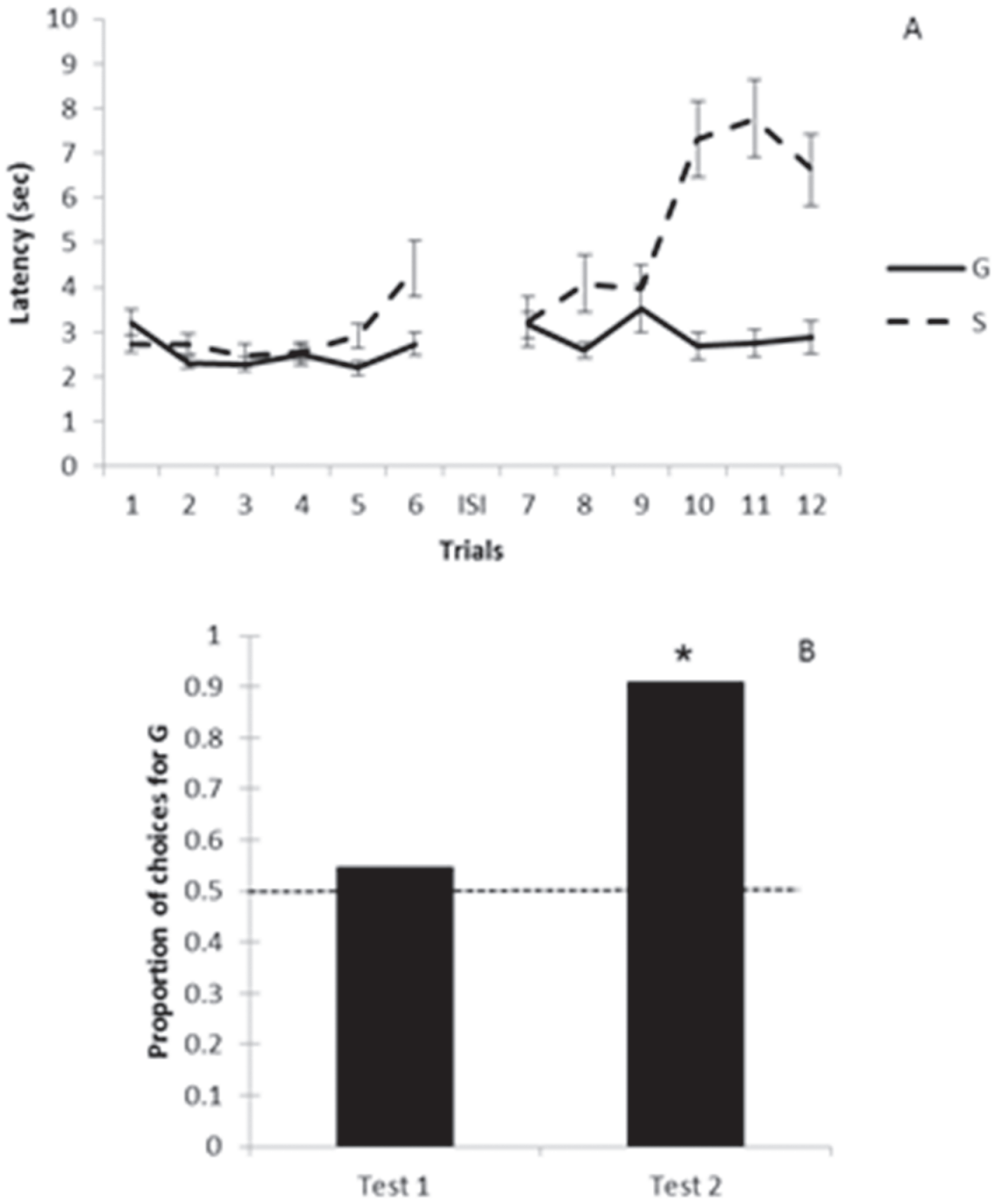
(A) Study 1: Mean latency as a function of trials with the generous and selfish experimenters. The dashed line represents the latency to approach the selfish (S) experimenter, whereas the full line represents the latency to approach the generous (G) experimenter. ISI: inter-session interval. Error bars denote ±1 sem. (B) Study 1: Proportion of choices for the generous (G) over the selfish (S) experimenter. The horizontal dashed line indicates the 0.50 chance level. *: p < 0.05

Fig. 3A shows the latencies to approach the bowl after the pointing gesture across training trials with G and S. We found no significant difference between the cumulative latency in trials with G against that in trials with S in the first training block (trials 1–6: Z = -1.49, N = 12, p = 0.136). In contrast, a similar analysis for the second training block (trials 7–12) showed that the cumulative latency of trials with G was significantly lower than that of trials with S (Z = -2.82, N = 12, p = 0.005). When we compared cumulative latencies between the first and the second training blocks (session 1–6 against sessions 7–12), we did not find any significant difference in sessions with G (Z = -0.31, N = 12, p = 0.75). But, the cumulative latency in the second training block was higher than in the first one in sessions with S (Z = -2.903, N = 12, p = 0.004; see Fig. 2A). Es’ gender had no significant effect on cumulative latencies (in trials with G: U = 12, Z = -0.96, n_men_ = 6, n_women_ = 6; in trials with S: U = 14, Z = -0.64, p = 0.52 n_men_ = 6, n_women_ = 6).

#### Performance in the pointing task

Considering the first and second blocks of trials together, dogs chose the bowl with food in 91.6% of the trials with G and in 76% of the trials with S. Both performances were significantly above the 0.50 chance level (for trials with G: *t*(11) = 25.72, p<0.0001; for trials with S: *t*(11) = 12.47, p<0.0001) and the difference between them was marginally significant (*t*(11) = 2.13, p = 0.056). Besides, there was 1 no-choice response out of 12 (8%) incorrect responses in trials with G, and 15 out of 34 (44%) in trials with S. This difference in the frequency of no-choice responses between Es was significant (Fisher’s exact test, p < 0.05).

Performances were not significantly different in trials with G than in trials with S when the first and second blocks of sessions were analyzed separately (first block: *t*(11) = 1.65, p = 0.95; second block: *t*(11) = 1.56, p = 0.14).

#### Preferences in choice tests

In the first test, one dog did not make a choice so it was discarded. Six of the 11 remaining dogs chose G and five chose S (Binomial test, N = 11, p = 0.22). In the second choice test, 10 animals chose G, 1 chose S and 1 did not make a choice. Therefore, as a group, dogs showed a preference for G over S in the second choice test (Binomial test, N = 11, p = 0.026; see Fig. 3B and Table 1). Concerning the time in proximity to each E during the first test, dogs spent a mean (±1 sem) of 3.82 (0.85) sec in proximity to G and a mean of 2.94 (0.67) sec in proximity to S; this difference turned out not to be significant (*t*(10) = 0.96, p = 0.35). We did not find any significant difference for the time in proximity to each E in the second choice task either (*t*(10) = 1.03, p = 0.32) or when we compared the time in proximity to G and S for the first versus the second choice test (for trials with G: *t*(11) = -0.37, p = 0.71; for trials with S: *t*(10) = -0.14, p = 0.88).

### Discussion

Results showed an increase in latencies between the first and the second block of trials when the pointing gesture was performed by the selfish experimenter. In contrast, latencies in trials with the generous experimenter did not change across blocks. In addition, dogs did not show a preference between experimenters in the first choice test, but preferred to approach the generous over the selfish experimenter in the second choice test. These results indicate that dogs learned to discriminate the experimenter’ generous and selfish attitudes, and developed a preference for the generous experimenter, though only after repeated interactions with both of them.

In terms of dogs’ performance during the pointing task, we found marginal evidence that they responded differently to the cues given by each experimenter. Indeed, performance in this task was above chance levels with both experimenters. This could be surprising particularly in the case of the selfish experimenter because subjects kept responding to his/her pointing gesture despite never getting the food from the bowl in those trials. Above-chance performance in trials with the selfish experimenter could be the result of at least two non-exclusive mechanisms. On one hand, it could be that dogs’ response to the pointing gesture takes longer to extinguish (than the number of trials ran in this study) because dogs are used to responding to these gestures successfully in their everyday lives [41]. Stable latencies across blocks for the generous experimenter, in contrast to the lengthening of latencies in trials with the selfish experimenter, agree with this hypothesis. In addition, we observed significantly more no-choice (extinction) responses in trials with the selfish than with the generous experimenter. On the other hand, it could be that the food given by the handler at the end of trials with the selfish experimenter maintained dogs’ responses by delayed reinforcement. In this sense, the response to the pointing gesture persisted, though at a lower performance level than the response in trials with the generous experimenter in which reinforcement for following the gesture was immediate. Nevertheless, unsystematic observations of dogs’ behavior suggest that, dogs were mostly focused on the experimenters and did not pay much attention to the handler during trials and choices. This observation is consistent with the fact that dogs almost always chose one of the experimenters in choice tests (23 out of 24 choice tests in study 1), instead of staying with the handler.

## Study 2

Since dogs required a high number of trials to develop a preference for the generous E in study 1, we ran a second experiment to evaluate if dogs’ discrimination between G and S could be enhanced. Given that dogs may behave differently towards a human stranger depending on his or her gender [37], we hypothesized that differences in the Es’ gender might improve the speed at which dogs learned the discrimination between G and S. With this hypothesis in mind, we ran a protocol identical to that of the previous study with the only difference that, for any given dog, Es were of different gender.

### Method

#### Subjects

We evaluated 22 naïve dogs. Six dogs were discarded: two because they showed signs of fear, three due to lack of motivation and one because it did not make a choice in any of the choice tests. The final sample consisted of 16 subjects of different breeds (1 Shih Tzu, 4 Golden Retrievers, 1 Boxer, 2 German Shepherd, 1 Border Collie and 7 mixed breeds), 10 females and 6 males. Mean (±1 SD) age was 5.26 (2.28) years old (see Table 1 for further details about individual subjects). All other conditions and criteria were identical to those of study 1.

#### Apparatus and procedure

The apparatus and procedure were identical to those described for study 1 with two exceptions: 1) for half of the subjects, the G role was played by a female E and the S role by a male E, and for the other half, it was the other way round; and 2) during choice tests, while standing, both Es held a bowl with food in their hands at the chest level; we introduced this modification to increase dogs’ motivation to approach the Es and elicit begging responses.

### Results

#### Latencies in training trials

**Fig 4:**
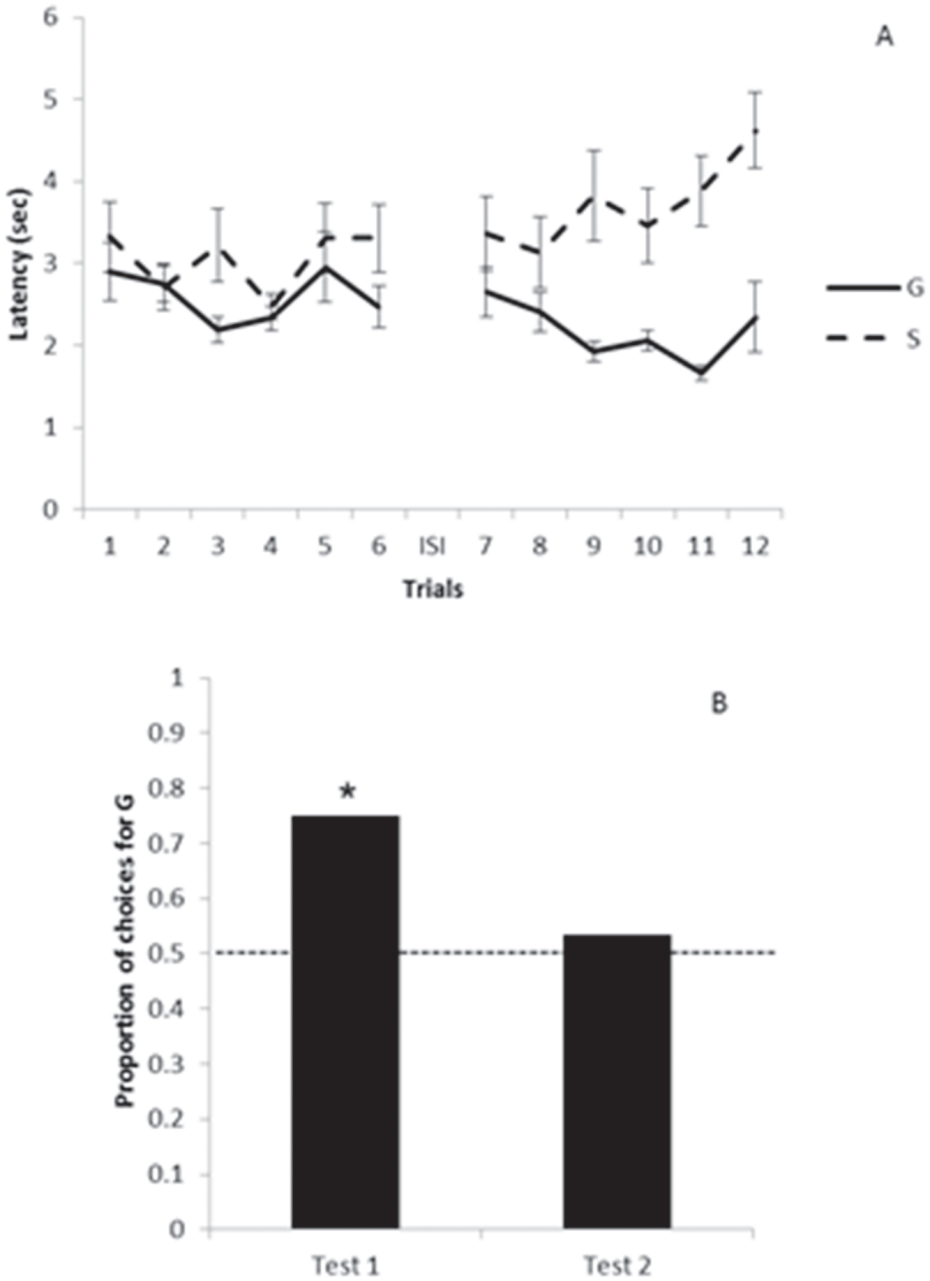
(A) Study 2: Mean latency as a function of trials with the generous and the selfish experimenters. The dashed line represents the latency to approach the selfish (S) experimenter, whereas the full line represents the latency to approach the generous (G) experimenter. ISI: inter-session interval. Error bars denote ±1 sem. (B) Study 2: Proportion of choices for the generous (G) over the selfish (S) experimenter. The horizontal dashed line indicates the 0.50 chance level. *: p < 0.05

The comparison of cumulative latencies between trials with G and S in the first training block shielded a marginal difference (Z = -1.91, N = 16, p = 0.056), which meant that dogs tended to make a faster approach towards the bowl in trials with G than in trials with S. In the second block, we found a significant difference in cumulative latencies as a function of whether the E was G or S in the same direction as before (Z = -3.51, N = 16, p = 0.0001). We did not find any significant difference between the cumulative latencies of the first and the second blocks for trials with G (Z = -1.65, N = 16, p = 0.098) or for trials with S (Z = -1.65, N = 16, p = 0.098; see Fig. 4A).

An analysis using the *Mann-Whitney U* test showed a non-significant difference in cumulative latencies as a function of Es’ gender (for trials with G, U = 22, Z = -1.05, p = 0.29, n_men_ = 8, n_women_ = 8; for trials with S, U = 23, Z = -0.94, p = 0.34, n_men_ = 8, n_women_ = 8).

#### Performance in the pointing task

Dogs chose the correct bowl in 91% of trials with G, and in 89% of the trials with S. Both performances were significantly higher than expected by chance (G: t(15) = 32.2, p<0.0001; S: *t*(15) = 22.23, p<0.0001). In contrast to study 1, the difference between them was not statistically significant (*t*(15) = 0.62, p = 0.53). However, dogs showed 2 no-choice responses out of 16 (12.5%) incorrect choices in trials with G, and 10 out of 21 (52.3%) in trials whit S. This difference in the frequency of no-choice responses between Es was significant (Fischer’s exact test, p < 0.05).

#### Preference in choice tests

In the first choice test, 12 out of 16 dogs chose G over S in their first approach (Binomial test, N = 16, p = 0.02). However, in the second choice test, only 8 animals chose G, 7 subjects chose S, and 1 animal did not choose (binomial test, N = 15, P = 0.19; see Fig. 4B and Table 1).

With regard to the Es’ gender, in the first choice test, 10 animals chose the woman and 6 chose the man (*X* ^2^_1_, p = 0.31), suggesting that animals chose as a function of the Es’ attitudes and not as a function of their gender. In the second choice test, 3 dogs chose the woman and 13 dogs chose the man (*X* ^2^_1_, p = 0.012). Only 6 of these 13 male Es had played the G role (x^2^_1_, p = 0.78). Indeed, six of the seven animals that chose the selfish man in the second choice test, had chosen the generous woman in the first choice test, suggesting that they might have switched after being non-reinforced in the initial test.

In terms of the time spent in proximity to each E in the first choice test, dogs spent a mean (±1 sem) of 7.92 (1.32) sec in proximity to G, and a mean of 1.91 (0.71) sec in proximity to S (*t*(15) = 3.29, p = 0.005). In the second choice test, dogs spent a mean of 5.00 (1.19) sec near G and a mean of 5.58 sec (1.35) near S (*t*(14) = 0.26, p = 0.79). Comparisons between the first and the second choice tests did not show any statistical difference for the time spent near G or S (for G: *t*(15) = 1.86, p = 0.081; for S: *t*(15) = -1.93, p = 0.072).

### Discussion

Results of study 2 indicate that dogs took longer to approach the bowl in selfish than in generous trials. This result is similar to what was found in study 1. Unlike the first study, however, there was no effect of the experimenters’ attitudes on the percentage of correct responses in the pointing task of study 2, although dogs showed more extinction (no-choice) responses with the selfish than with the generous experimenter.

In study 2, dogs successfully discriminated between experimenters and showed a preference for the generous over the selfish person with only six training trials. The “preference” for the generous experimenter in the first choice test was detected with the two dependent variables registered (i.e., the first choice and the time in proximity to each E). This contrasts with results of study 1 in which dogs did not show a preference in the first choice test, thus suggesting that the difference in experimenters’ gender improved dogs’ learning speed in the discrimination task in study 2.

## Study 3

The recognition of other animals could be performed at different levels of discrimination, from recognition of the species, through recognition of social categories such as a hierarchy level, to the recognition of particular individuals [42]. Indeed the ability to recognize individuals might be a pre-requisite for the attribution of reputation [13]. Considering that dogs’ performance improved from study 1 to study 2, we wondered whether dogs recognized each E individually or instead only discriminated between the Es based on their gender. According to this last idea, dogs could have followed a simple rule such as “woman = generous/man = selfish”, or the other way round. To answer this question, we conducted a third experiment in which the female and the male Es were changed between the training block and the choice test. In other words, Es left the room after training trials, and two new Es (a woman and a man) came back for the choice test. To control for the fact that Es left the room, we had a control group in which Es also left the room, but the same Es came back for the choice test (i.e., Es were not changed in the control group).

If dogs’ learned to discriminate between Es based only on their gender, we would expect dogs in the experimental treatment to choose the changed E whose gender matched that of the generous E of training trials. Otherwise, it would mean that dogs used gender and at least another cue particular to the individual E, thus showing a skill closer to individual recognition.

### Method

#### Subjects

We recruited 30 naïve dogs for this study. We discarded 7 dogs, one because it did not make a choice in the choice test, two due to lack of motivation for food, one due to interruptions of the owners during the protocol, two for showing signs of fear, and one due to errors during the procedure. We ended up with a sample of 23 dogs of diverse breeds (1 Bichon Frisé, 1 French Bulldog, 2 Labrador Retrievers, 2 Boxer, 2 Pitbulls, 1 German Shepherd, 2 Poodle, 1 Siberian Huskie, 1 Rottweiler, 1 Belgium Shepherd, 1 Setter, 1 Border Collie, 1 Golden Retriever, and 6 mixed breeds), 7 females and 16 males. Mean (±1 SD) age was 4.47 (2.17) years old (see Table 1 for further details of individual subjects). All other conditions and criteria were identical to those described for the two previous studies.

#### Apparatus and procedure

The apparatus and procedures were identical to those described for study 2 with three exceptions: 1) the G role was always played by a woman and the S role by a man; this was done to facilitate the task given that previous studies have shown that dogs display more fear and aggression towards men than women [37, 43]; 2) in the experimental group, after the first training block, both Es left the room and, after 1 min, two new Es (a woman and a man) unknown to the subjects entered the room for the choice test; in the control group, the same Es that left the room, after 1 min, re-entered the setting for the choice test; and 3) unlike the two previous studies, this protocol consisted of only one session of training trials with each E and, after that, a single choice test.

The role of the control condition in study 3 was to maintain constant between groups the fact that Es left and re-entered the experimental room. This was implemented to control for the possibility that the experimenters’ movements out of and into the room had an effect on dogs’ memory for the experiences in training trials.

### Results

#### Latencies in training trials

**Fig 5:**
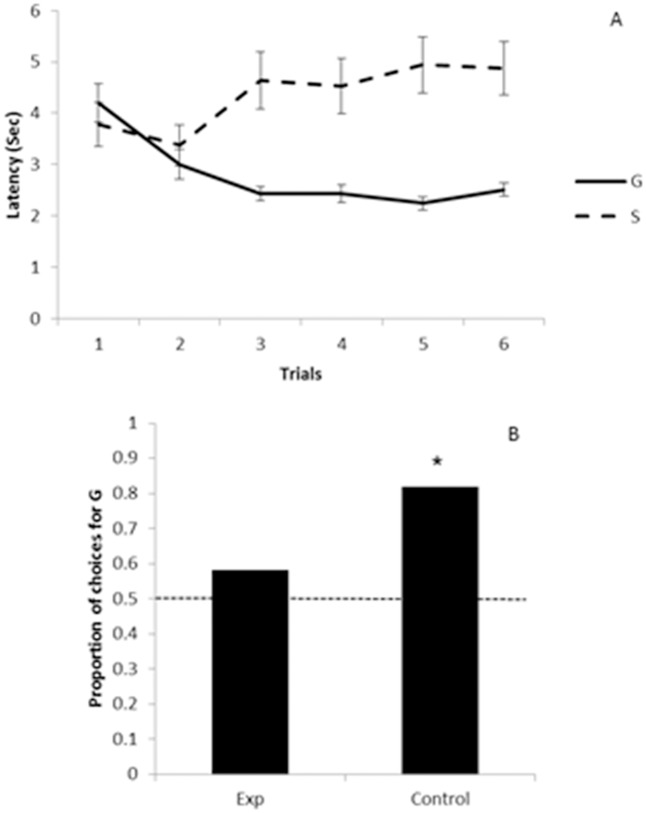
(A) Study 3: Mean latency as a function of trials with the generous and selfish experimenters. The dashed line represents the latency to approach the selfish (S) experimenter, whereas the full line represents the latency to approach the generous (G) experimenter. ISI: inter-session interval. Error bars denote ±1 sem. (B) Study 3: Proportion of choices for the generous (G) over the selfish (S) experimenter. The horizontal dashed line indicates the 0.50 chance level. *: p < 0.05.

Fig. 5A shows mean latencies in trials with G and in trials with S, pooling together the data from the experimental and the control groups (protocols of both groups were identical at that point). As expected, we did not find any difference in latencies when we compared the experimental group against the control group neither in trials with S nor in trials with G (*Mann-Whitney U test*; G: U = 50. Z = -0.98, p = 0.32, n_experimental_ = 12, n_control_ = 11; S: U = 63, Z = -0.18, p = 0.85, n_experimental_ = 12, n_control_ = 11). We neither find any significant difference when we compared trials with G against trials with S of the pooled data (*Wilcoxon Matched Pairs test*, Z = -1.43, N = 23, p = 0.15).

#### Performance in the pointing task

In the experimental group, the dogs chose the bowl with food in 87.5% of the trials with G and in 69.4% of the trials with S. Both performances were significantly above the 0.50 chance level (trials with G: *t*(11) = 21.82, p<0.0001; trials with S: *t*(11) = 6.52, p = 0.0001). In the control group, dogs chose the bowl with food in 87.8% of the trials with G and in 72.7% of the trials with S. These performances were also significantly above the 0.50 chance level (trials with G: *t*(10) = 20.13, p<0.0001; trials with S: *t*(10) = 7.86, p<0.0001).

Taking together the number of no-choice responses in relation to the number of incorrect responses in both groups, we registered 1 out of 17 (5.88%) in trials with G, and 20 out of 38 (52.6%) in trials with S. This difference in no-choice responses between Es was significant (Fisher’s exact test, p < 0.05).

We also did a repeated-measures ANOVA of dogs’ performance with experimental/control group as a between-subject factor and G/S trials as a within-subject factor. We found a main effect of Es’ attitude, meaning that dogs showed a higher performance level in trials with G than in trials with S (F_1, 21_ = 6.85, p = 0.016), but we did not find any effect of group (F_1,21_ = 0.64, p = 0.803) or of the interaction between group and attitude (F_1,21_ = 0.052, p = 0.82).

#### Preference in the choice test

In the experimental group, 5 of the 12 subjects chose the E that had replaced G, whereas 7 subjects chose the E that had replaced S (Binomial test p = 0.19, n = 12). In the control group, 9 of the 11 subjects chose G, whereas only 2 subjects chose S, thus showing a significant preference for the G over S (Binomial test p = 0.026, n = 11) (see Fig. 5B and Table 1). In terms of the time in proximity to each E, animals spent a mean (±1 sem) of 6.53 (1.14) sec near the replacement of G and 5.73 (1.26) sec near the replacement of S in the experimental group (*t*(11) = 0.35, p = 0.73). In the control group, dogs spent 7.09 (1.25) sec near G and 2.59 (1.15) sec near S (*t*(10) = 2.43, p = 0.035).

### Discussion

Alike the first block of sessions in the two previous studies, dogs’ latencies to approach the bowl did not differ as a function of the experimenters’ attitudes in study 3.

Yet we found that dogs’ performance in the pointing task was higher in trials with the generous experimenter relative to trials with the selfish person. Similarly to results from study 2, control dogs showed a significant preference for the generous over the selfish experimenter. On the contrary, experimental dogs’ choices did not reveal any systematic preference between the experimenters who replaced the generous and the selfish trainers. This lack of preference in the experimental group suggests that the preference seen in the control group as well as that seen in study 2 did not rely on dogs using a simple rule such as “women = generous/men = selfish”, or vice versa. Therefore, these results seemingly indicate that, besides gender, dogs codified one or more other distinctive characteristic of each person. This is evidence of an ability that comes closer to individual recognition.

## General Discussion

Results from present studies show that domestic dogs could recognize generous and selfish people. This discrimination was learned through direct interactions with previously unknown humans in a communicative task, and was later remembered and expressed as a systematic preference in choice tests. In this context, dogs’ attribution of reputation involved, first, the discrimination of generous and selfish attitudes, and second, the association of each attitude with the corresponding individual. We argue that the fact that dogs developed a preference for the generous over the selfish experimenter could have involved the individual recognition of each person.

In the current protocols, differences between experimenters’ attitudes involved not only differences in the provision of food but also on the use of ostensive cues (e.g., alternating the gaze between the correct bowl and the dog’s eyes) and social reinforcement (e.g., talking to the dog in a nice as opposed to a neutral tone of voice). In this sense, experimenters acted as complex stimuli. The selfish experimenter made the proximal gesture towards the food, but did not allow the dog to eat, thus avoiding any reinforcement. In the case of the generous experimenter, his or her ostensive cuing could have facilitated the direction of dogs’ attention towards the communicative task, thus enhancing learning [44]. Social reinforcement by the generous experiment could have improved the discrimination between experimenters as well.

These differences between the generous and the selfish experimenters all went in the same direction in present studies, namely making the generous experimenter a nicer target for dogs to choose. However, we did not intend to discriminate among the efficacy of the different cues provided. Therefore, we cannot conclude about the relative effect of food reinforcement, social reinforcement, and the use of ostensive cues in the present protocols. Previous data from the literature in this respect are also inconclusive. For example, Freidin et al. [31] found that dogs discriminated between demonstrators when the beggar who asked for food presented both verbal and gestural cues expressing satisfaction or dissatisfaction with their response; however, dogs did not show a preference between demonstrators when only verbal or gestural cues were used by the beggar. On the contrary, Marshall Pescini et al. [34] and Petterson et al. [40] observed that verbal cues had a stronger impact than gestural cues in a similar social discrimination task. Future studies should focus on isolating the effect of the different cues and rewards used in the present studies to assess their relative efficacy on dogs’ discrimination and tracking of human behavior.

Another issue with present results is that they do not allow us to distinguish whether dogs preferred the generous experimenter or, instead, avoided the selfish person in choice tests. More troublesome for the present goals is the possibility that dogs’ behavior may have been influenced by the handler’s food reinforcement in trials with the selfish experimenter. Fortunately, various pieces of evidence suggest that dogs’ behavior in choice trials was not affected by prior reinforcement by the handler. First, considering all choices taken together from the three studies reported, dogs did not make a choice between the experimenters and stayed with the handler in only 3 cases out of 79 choice tests, which is a negligible frequency. Second, the increase in the latency to approach the selfish experimenter as well as the higher frequency of no-choice responses in training trials with the selfish experimenter could be interpreted as indicating the progressive extinction of his/her unreinforced signaling gesture. Similar findings have been reported in the extinction phase of a standard signaling task with dogs [41]. Last, unsystematic observations during training trials and choice tests suggest that dogs did not pay much attention to the handler, but were focused on the experimenters, even in training trials with the selfish experimenter in which food reinforcement was only provided by the handler.

One of the main findings in the present set of studies was that dogs solved the task faster when they could rely on the experimenters’ gender to discriminate between them. Results from study 2 showed that dogs developed a preference after only six trials with each experimenter as recognition was facilitated by using experimenters of different gender. This is consistent with previous findings showing that dogs react differently to men and women [37, 43]. Ratcliffe et al. [45] showed that dogs are even capable of associating an unknown recorded (male or female) voice with a man or a woman. These authors used the preferential looking paradigm in which animals look longer to events that violate their expectations. Accordingly, dogs looked longer towards the incorrect individual whose gender did not match the gender of the voice (e.g., female voice in the presence of a man) as measured both by their first gaze as well as by the total gaze time [45]. Interestingly, the ability to discriminate men and women is not exclusive of dogs. It has been recently reported that laboratory rats and mice display stronger stress responses in the presence of male relative to female experimenters [46]. Even more, results from the present study 3 showed that dogs used the experimenters’ gender not simply to create a general rule, such as “women = generous/men = selfish” or vice versa, but to recognize the physical characteristics of each experimenter. This improvement in learning when the experimenters’ gender served as a discriminatory cue suggests that (lack of) discrimination of the experimenters was limiting dogs’ performance in study 1. In this sense, it seems likely that dogs discriminated more rapidly between the different attitudes than between individuals. This increased difficulty in associating certain consequences with a specific person thus points in the direction of the intricacy of individual recognition.

The capacity to recognize individuals is a key adaptation in many social species [47] and has been particularly studied in the context of agonistic interactions in different organisms, from insects to birds and mammals [48–50]. As suggested by experiments, the process of individual recognition requires numerous interactions, and, in many circumstances, does not happen spontaneously, not even in humans. For example, in a “blind to change” study on the field, Simons and Levin [51] sent their actors to the street where the following scene was displayed: one of the actors would stop a person (the participant), present a map and ask for directions; while they were talking, two other actors carrying a door walked through the middle of the conversation, and the original actor who had been talking to the participant swapped with one of the actors that was carrying the door. The surprising finding was that approximately 50% of participants failed to notice that they were talking to a different person after the switch [51], thus suggesting that humans may also struggle to recognize and remember unfamiliar faces. In nonhuman animals, individual recognition of conspecifics in other species has only been shown in protocols involving several interactions. For instance, Lai and Johnston [48] exposed two male hamsters to three mutual encounters which lasted until one of them escaped or displayed submissive behaviors. Afterwards, losers were capable of recognizing the winner’s odor and could even discriminate it from the odor of other dominant males. This ability to recognize individuals may be especially crucial when interactions can result in aggression. Such a potent unconditional stimulus (e.g., physical pain or harm, or the threat of it) may facilitate discrimination and later recall of individuals.

Some authors claim that individual recognition may play an important role in cooperative interactions as well [8], and there is information that the amount of experience with each individual is an important factor influencing performance in this context too. For example, Freidin et al. [31] showed that dogs could discriminate people’s (dis)satisfaction reactions after watching just three interactions of each demonstrator with a third person. However, dogs did not individually recognize the demonstrators, but instead, used the demonstrator’s place in demonstration trials (left or right) as a cue to later guide their choices (i.e., local enhancement). These results are in agreement with those recently reported by Nitzschner et al. [35]. The only study in which dogs were able to solve the task even after experimenters controlled for local enhancement was the study done by Kundey et al. [33], in which dogs watched a large number of interactions (10 with each demonstrator) before they could choose. In any case, it is important to remark that local enhancement was not a worry in present studies because experimenters were at the center of the stage in training trials, whereas they stood on a side (left or right) for choice tests.

The evidence for the features and factors that may improve dogs’ discrimination of individuals is scant, and it has been mainly studied in protocols with familiar persons. For example, Racca et al. [52] found that dogs could rely on visual information to discriminate between familiar and unfamiliar faces. Moreover, dogs were able to recognize whether the match between a face and a voice was correct when dealing with familiar individuals [53]. This literature may lead us to think that dogs were presented with a very difficult task in the present studies given that they were exposed to unfamiliar individuals. In study 1, not only were the experimenters unfamiliar but they were also of the same gender. When gender was an extra differentiating feature between experimenters in studies 2 and 3, dogs’ discrimination clearly improved. In addition, it is possible that the use of a very salient cue such as the ostensive proximal pointing gesture had an overshadowing effect upon the experimenter’s individual characteristics. In this sense, dogs may have paid attention only to the experimenter’s arm and hand in the first few trials, neglecting other more distinguishing features. Most likely, hands were an insufficient cue to discriminate the experimenters in the choice test, which would explain why dogs did not immediately show a preference between demonstrators in study 1. Future efforts could be directed at assessing dogs’ differential attention to specific parts of the human body and to other cues present in the object choice task to gather independent evidence of a potential overshadowing phenomenon.

Finally, a number of other possible factors could have, in principle, affected our results beyond the experimenters’ differential attitudes. Fortunately, several controls help us discard these alternatives. To begin with, dogs’ age and size had no apparent effect on the latency to approach the bowl in training trials, on subjects’ performance in the signaling task, or on any behavior in choice tests. Besides, it was possible that dogs had a gender bias in choice tests of studies 2 and 3 based on their owners’ gender. This was, however, unlikely given that most dogs (82%) lived with a woman and a man in the same home. In addition, previous data from the literature shows that dogs tend to react differently to men and women, independently of their owners’ gender [45, 53–55]. Last but not least, the fact that dogs learned the task independently of their owners’ gender in the present studies clearly shows that dogs were mainly guided by the experimenters’ attitudes and not their gender.

In sum, we showed that dogs could discriminate generous from selfish attitudes, associate such behaviors with specific humans, and later use that information to decide who to approach in a choice test. The amount of experience with each person as well as people’s gender both affected dogs’ performance. In the end, dogs may use the ability to individually recognize people to predict who is more likely to provide access to valuable resources, which seems very advantageous given that domestic dogs depend much on humans.
